# Expansion of the *Candidozyma haemuli* Species Complex - The Novel Species *Candidozyma molenica,* Isolated from Clinical and Environmental Sources

**DOI:** 10.1007/s11046-025-00985-z

**Published:** 2025-09-01

**Authors:** Annemarie Zandijk, Tjomme van der Bruggen, Matthias Sipiczki, Wim J. E. Tissing, Tom F. W. Wolfs, Bert Gerrits van den Ende, Marizeth Groenewald, Ferry Hagen

**Affiliations:** 1 Department of Medical Mycology, Westerdijk Fungal Biodiversity Institute (WI-KNAW), Uppsalalaan 8, 3584 CT Utrecht, The Netherlands; 2 Department of Medical Microbiology, University Medical Center Utrecht, Utrecht, The Netherlands; 3 Department of Genetics and Applied Microbiology, University of Debrecen, Debrecen, Hungary; 4 Princess Máxima Center for Pediatric Oncology, Utrecht, The Netherlands; 5 Wilhelmina Children’s Hospital, Utrecht, The Netherlands; 6 Institute for Biodiversity and Ecosystem Dynamics, University of Amsterdam, Amsterdam, The Netherlands

**Keywords:** *Candidozyma auris*, *Candidozyma haemuli* species complex, *Candidozyma khanbhai*, *Candidozyma vulturna*, Emerging pathogen, Misidentification

## Abstract

Due to the recent unprecedented global rise of *Candidozyma auris* in hospital environments the members of the *Candidozyma haemuli* species complex have raised significant interest of clinicians and researchers. Until the finding of *C. auris*, the species complex did not receive much attention as the known pathogenic species were only rarely encountered in hospitals and clinical diagnostic laboratories. During the past years several new species were described, such as *Candidozyma khanbhai* and *Candidozyma vulturna*, that were found to be of clinical importance. Here, we used phylogenetic and phenotypic analyses –including antifungal susceptibility testing– to characterize and describe a new and potentially clinically relevant yeast that we obtained from clinical specimen and flowers, representing the proposed novel species *Candidozyma molenica*.

## Introduction

The highly polyphyletic ascomycetous genus *Candida* accommodates a wide variety of yeast species classified into nearly twenty defined clades [1, 2]. Among them are clades that include well-known human pathogenic yeasts, like the *Lodderomyces*-clade that includes the *Candida* senso stricto species *Candida albicans*, *Candida parapsilosis* and *Candida tropicalis*, the *Pichia*-clade that houses *Pichia kudriavzevii* (=*Candida krusei*) and *Pichia norvegensis* (=*Candida norvegensis*), the *Nakaseomyces*-clade that includes *Nakaseomyces glabratus* (=*Candida glabrata*), *Nakaseomyces bracarensis* (=*Candida bracarensis*) and *Nakaseomyces nivariensis* (=*Candida nivariensis*) [1–3]. The *Metschnikowiaceae*-clade did not receive much attention from the clinical diagnostic field, despite that it accommodates *Clavispora lusitaniae* (previously *Candida lusitaniae*), a relative common cause of infection, and the *Candidozyma haemuli* species complex [3].

The relative obscurity of the *C. haemuli* species complex changed drastically with the emergence of the multidrug resistant *Candida auris*, a species described fifteen years ago that soon thereafter appeared to be involved in numerous difficult to control nosocomial outbreaks [4]. The rapid emergence of *C. auris* has astounded medical mycologists and clinicians, its nosocomial dispersal went often unnoticed as the culprit was not identified or misidentified as another—often antifungal susceptible—species [5]. *Candida auris* is a member of the *Candida haemulonii* species complex that forms a basal group within the *Metschnikowiaceae*-clade and is closely related to the genus *Clavispora* [6, 7]. Recently, a proposal was made to reassign the members of the *Candida haemulonii* species complex to the genus *Candidozyma,* also belonging to the order *Serinales* [8]. This species complex contains, next to *C. auris*, other human pathogens, such as *Candidozyma haemuli*, *Candidozyma duobushaemuli*, *Candidozyma pseudohaemuli*, *Candidozyma vulturna* and the recently described species *Candidozyma khanbhai* [6, 9, 10]. Notably, *C. vulturna* was recently found to be involved in a long-lasting outbreak in China and an outbreak in Brazil [11, 12]. Here we describe a new species that was isolated from clinical specimens in the Netherlands and from environmental sources in Belize, this novel species within the *Candidozyma haemuli* species complex is here described as *Candidozyma molenica* sp. nov.

### Case Description

A two-year old male patient was diagnosed with a solid tumor in the Princess Máxima Center for Pediatric Oncology (Utrecht, The Netherlands). During the first few days after admission, before the diagnosis was established, swabs (Sigma Transwab; Beldico, Breda, The Netherlands) were taken from the throat and rectum for fungal and bacterial culturing to determine the colonization status as part of the routine workup for all pediatric oncology patients. Culturing of the fungi were performed at the Department of Medical Microbiology of the University Medical Center Utrecht, Utrecht, The Netherlands, using malt extract agar (3% w/v; Oxoid, Basingstoke, United Kingdom) containing chloramphenicol 25 mg/L, colistin 15 mg/L and vancomycin 6 mg/L. After two days of incubation at 35 °C, a yeast grew from both throat and rectum swabs. Subsequent identification was performed by matrix-assisted laser desorption ionization–time of flight mass spectrometry (MALDI-TOF MS; Bruker MALDI Biotyper system, BDAL-8468 database; Bruker Daltonics, Bremen, Germany). The MALDITOF-MS results suggested *Candidozyma pseudohaemuli* (=*Candida pseudohaemulonii*), but identification was not robust with score values ranging between 1.5 and 1.7. In addition, antifungal susceptibility testing according to EUCAST was performed (see below). Subsequently, the strains were sent to the Westerdijk Fungal Biodiversity Institute (Utrecht, The Netherlands) for further analyses as described below. During the following eleven months no suspicion of fungal infection was documented in the electronic patient file and no antifungals were administered. Fungal growth from a throat swab six months later remained negative. Therefore, the initial presence of the yeast in the throat and rectum was considered temporary colonization.

## Material and Methods

### Strains and Media

The two clinical strains CBS 18490^T^ (throat swab) and CBS 18491 (rectal swab), obtained from a single patient, were subcultured onto 2% glucose, 0.5% yeast extract, 1% peptone, and 1.5% technical agar (GYPA) medium, and incubated for 48 h at 35 °C. Additionally, type-strains from the recognized species in the *C. haemuli* species complex were obtained from the CBS Culture Collection hosted at the Westerdijk Fungal Biodiversity Institute, Utrecht, The Netherlands, and included *C. auris* CBS 10913^T^, *C. duobushaemuli*, CBS 7798^T^, *C. chanthaburiensis* CBS 10926^T^, *C. haemuli* CBS 5149^T^, *C. heveicola* CBS 10701^T^, *C. khanbhai* CBS 16213^T^, *C. konsanensis* CBS 12666^T^, *C. metrosideri* CBS 16091^T^, *C. ohialehuae* CBS 16092^T^, *C. pseudohaemuli* CBS 10004^T^, *C. ruelliae* CBS 10815^T^, and *C. vulturna* CBS 14366^T^. Additionally, *Cl. lusitaniae* CBS 6936^T^ was obtained to serve as phylogenetic basal lineage, and the *C. auris* strains AR0383, AR0385, AR0383 and AR1097 served as additional representatives of the various recognized lineages within this medically important species (strains obtained from the CDC Isolate Bank, Atlanta, GA, USA). The strains *Candida albicans* CBS 562^NT^, *Candida tropicalis* CBS 94^T^, *Nakaseomyces glabratus* CBS 138^T^, *Pichia kudriavzevii* CBS 573^T^, *Candida parapsilosis* CBS 604^T^, and *Candida orthopsilosis* CBS 10906^T^ were included to serve as culture controls and represent the most commonly found yeast in clinical diagnostic settings.

### DNA Sequencing and Phylogenetic Analyses

Genomic DNA was obtained using an in-house cetyltrimethylammonium bromide-based extraction protocol, as previously published [13]. Subsequently, the internal transcribed spacer (ITS) regions spanning ITS1-5.8S-ITS2 and the D1/D2 region of the large subunit ribosomal DNA were amplified using the primers ITS1 (5′-TCCGTAGGTGAACCTGCGG-3′) and ITS4 (5′-TCCTCCGCTTATTGATATGC-3′) for the ITS region, and NL1 (5′-GCATATCAATAAGCGGAGGAAAAG-3′) plus NL4 (5′-GGTCCGTGTTTCAAGACGG-3′) for the D1/D2 LSU target [14]. Bidirectional Sanger amplicon sequencing was performed with the same primers and the BigDye v3.1 chemistry (Applied Biosystems, Palo Alto, CA, USA). Raw data was generated using the ABI 3700xL Genetic Analyzer platform (Applied Biosystems) and output was manually checked and corrected using the SeqMan module as part of the Lasergene v17 software package (DNASTAR, Madison, WI, USA).

**Fig. 1:**
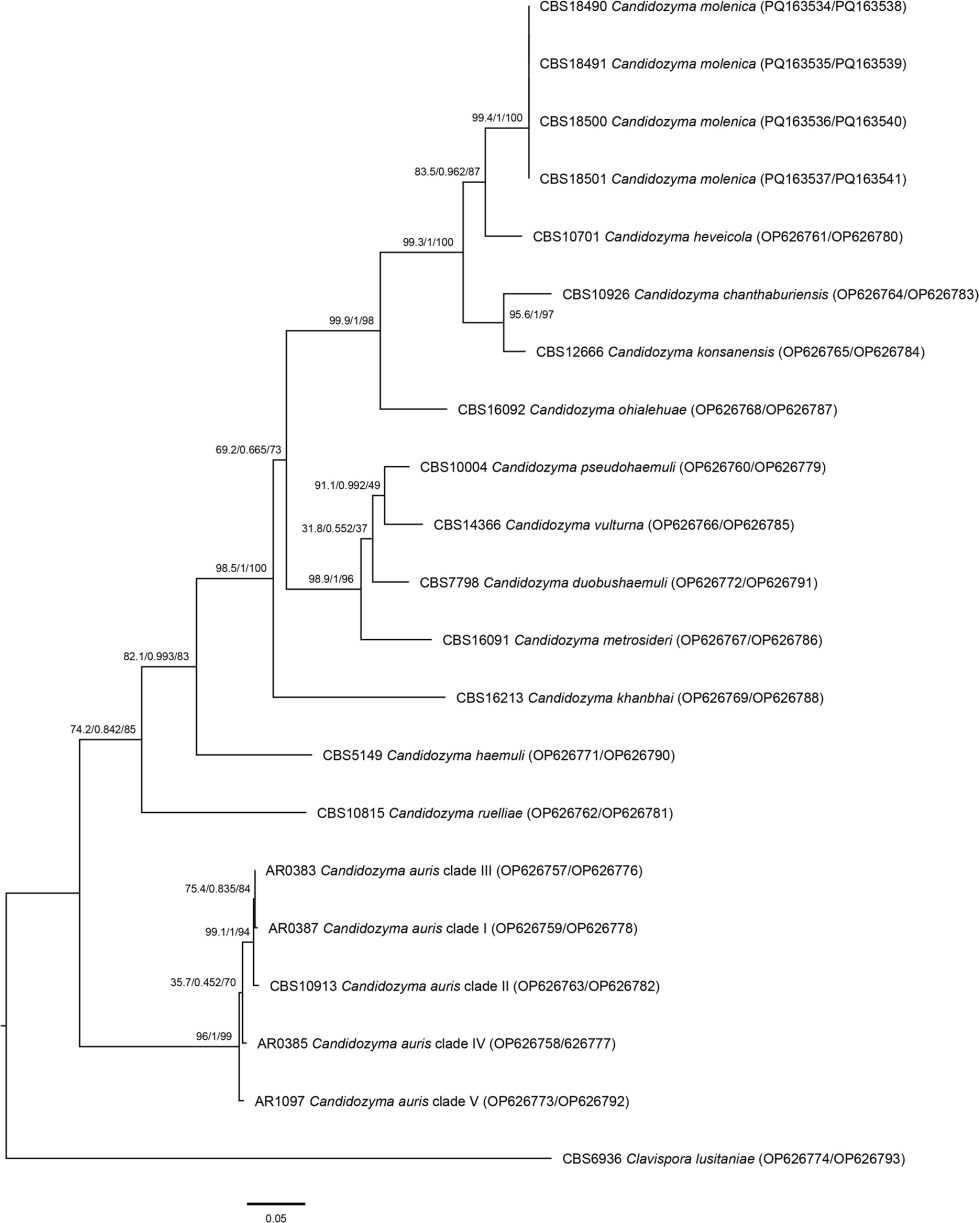
Phylogenetic analysis of the *Candidozyma haemuli* species complex. Sequences were aligned with MAFFT prior to creating a phylogenetic tree using IQ-tree with 1000 bootstrap repeats. The selected model of substitution is TNe + G4. The support values near the branches show likelihood ratio test/bayesian inference/bootstrap in this order

A BLAST search using the curated ISHAM ITS Sequence Database [15] resulted in < 97% similarity hit with *C. auris* and < 90% with *C. haemuli*. However, when using the NCBI Nucleotide database, a > 99% BLAST hit was observed with the strains 11–1116 and 11–1117 isolated in 2006 from unidentified flowers within a very mixed road-side population of plants in Belize City, Belize (17°30′30.5″ N, 88°11′15.6″ W). These two environmental strains were stored at the Department of Genetics and Applied Microbiology, University of Debrecen, Debrecen, Hungary. Both strains were obtained and underwent the same procedures to obtain the ribosomal DNA sequences as described above. Sequences were deposited in the NCBI Nucleotide database under accession numbers PQ163534–PQ163541, as indicated in Fig. 1.

Phylogenetic analyses were performed using the same dataset as used for the recent species description of *C. khanbhai*, which belongs to the same species complex as the four strains we investigated in the present study [9]. The alignment was made with MAFFT v7.409 using FFT-NS-i settings and visually checked and manually corrected prior phylogenetic reconstruction using 1000× bootstrapped Maximum Likelihood with the Tamura-Nei model—Gamma Distributed as determined by IQ-tree v1.6.4 as best fitting substitution model, with alignment-gaps treated as complete deletions and a nearest-neighbor-interchange as heuristic method using IQ-tree v1.6.4 with parameter settings: -redo -nt AUTO -nm 1000 -m MFP + MERGE -abayes -alrt 1000 -bb 1000 with substitution model TNe + G4 and 1000 bootstrap repeats to generate a phylogenetic tree based on the likelihood ratio test, bayesian inference, and bootstrap methodology [16, 17].

The strain obtained from the throat swab (2MG-A1904-10) and rectal swab (2MG-A1904-11) were deposited in the CBS Culture Collection under accession numbers CBS 18490^T^ and CBS 18491, respectively. The two strains isolated from flowers (11–1116 and 11–1117) were deposited under accession numbers CBS 18500 and CBS 18501, respectively.

### Phenotypic and Physiological Examination

**Fig. 2:**
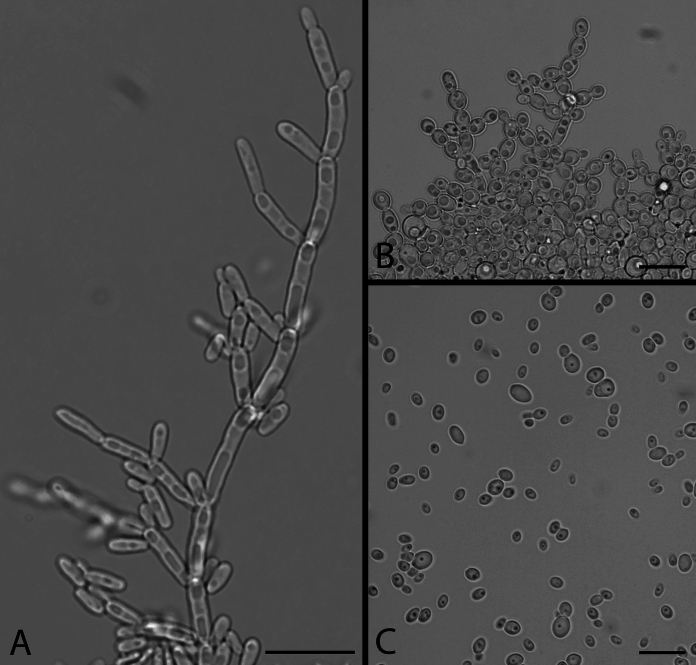
Phenotypic characteristics of *Candidozyma molenica*. *Candidozyma molenica* sp. nov. CBS 18490^T^. Slide cultures, showing the formation of pseudohyphae after 4 days at 25 °C onto GYPA (Panel A) and after 8 days at 37 °C onto GYPA (Panel B). Vegetative cells grown on GYPA for 3 days at 25 °C (Panel C). Bar, 10 µm

The colony morphology of the four strains was determined using plate cultures grown on 2% Glucose, 0.5% Yeast extract, 1% Peptone, and 1.5% agar (GYPA) for seven days at 25 °C. Microscopic pictures were taken, using an Axioskop 2 plus (Carl Zeiss, Jena, Germany) microscope fitted with a Nikon DS-Ri2 microscope camera (Nikon Instruments, Melville, NY, USA), of cells grown on GYPA at 25 °C for three days (Fig. 2c), and in addition of pseudohyphae using slide cultures after four days at 25 °C (Fig. 2a) and eight days at 37 °C (Fig. 2b) on GYPA [18]. Additionally, the mating compatibility of the four strains was tested by mixing them together on GYPA, Malt-Extract Agar (MEA; Oxoid, Basingstoke, United Kingdom), V8-juice agar (V8), Yeast extract-malt extract agar (YMA), and yeast carbon base plus 0.01% ammonium sulfate and 1.5% agar (YCBAS) plates and incubated at 25 °C for up to 12 weeks [18]. Fermentation and assimilation of carbohydrates was performed using API32C strips (bioMérieux, Marcy-l'Étoile, France) and incubated at 25 °C for up to 14 days. Assimilation of nitrogen compounds was assessed with the auxanographic method [18].

As chromogenic culture media are commonly used in clinical diagnostics, and that the here investigated strains are closely related to human pathogenic yeast species, the growth onto CHROMagar Candida and CHROMagar Candida Plus (CCP; CHROMagar, Paris, France) was determined. We inoculated strains CBS 18490^T^, CBS 18491, CBS 18500, and CBS 18501 onto these media together with the type-strains of *C. auris* (CBS 10913^T^), *C. haemuli* (CBS 5149^T^), *C. duobushaemuli* (CBS 7798^T^), *C. pseudohaemuli* (CBS 10004^T^), *C. vulturna* (CBS 14366^T^). The inoculated media were incubated at 35 °C for 48 h.

### Antifungal Susceptibility Testing

Antifungal susceptibility testing was performed according to the microdilution broth methodology as detailed in the EUCAST definitive document E.DEF 7.3.2 with growth measured as absorbance at a wavelength of 530 nm [19]. Antifungals included were 0.125–4 mg/L amphotericin B (Santa Cruz Biotechnology, Santa Cruz, CA, U.S.A.), 0.125–64 mg/L 5-fluorocytosine (Honeywell Fluka, Charlotte, NC, U.S.A.), 0.125–16 mg/L fluconazole (Pfizer, New York, NY, U.S.A.), 0.03–16 mg/L itraconazole (Santa Cruz Biotechnology), 0.03–16 mg/L voriconazole (Pfizer), 0.015–8 mg/L posaconazole (MSD, Haarlem, The Netherlands), 0.008–4 mg/L anidulafungin (Pfizer) and 0.008–4 mg/L micafungin (Cayman Chemical, Ann Arbor, MI, U.S.A.).

### MALDI-TOF MS Reference Spectra

Nowadays MALDI-TOF MS is a cornerstone diagnostic tool in clinical laboratories, but this rapid and cost-effective identification approach depends on the species reference spectra database. To enable the identification of the here proposed species *Candidozyma molenica*, a reference profile was created according to the manufacturer’s protocol (Bruker Daltonics).

## Results

### Phylogenetic Analysis

The combined phylogenetic analysis of the ITS and D1/D2 fragment of the LSU showed that CBS 18490^T^, CBS 18491, CBS 18500 and CBS 18501 had identical sequences and clustered closely to *Candidozyma heveicola* CBS 10701^T^ (Fig. 1). The 412 bp ITS sequence between the former four strains versus *C. heveicola* CBS 10701^T^ differed at 53 positions (12.7% variability), while for the 523 bp D1/D2 sequence this was only 9 sites different (1.7% variability). Phylogenetic analyses showed strong-to-full support values of 83.5–99.4 for the likelihood ratio test, 0.962–1 for the Bayesian inference, and 87–100 bootstrap support (Fig. 1). These five strains are all part of the previously recognized ‘environmental cluster’ of the *C. haemuli* species complex, as it contains species that were not reported from clinical sources, which includes *C. chanthaburiensis*, *C. konsanensis* and *C. ohialehuae* [20]. This environmental cluster is most closely related to species that are commonly, or even exclusively, known from clinical specimen, namely *C. duobushaemuli*, *C. khanbhai*, *C. metrosideri*, *C. pseudohaemuli*, *C. vulturna*. *C. haemuli* and *C. ruelliae* are both basal to all these species, while *C. auris,* represented by five strains—representing the five recognized clades—seems to be the basal species of the *C. haemuli* species complex when *Clavispora lusitaniae* is taken as an outgroup (Fig. 1).

### Phenotypic Characterization

The four studied strains CBS 18490^T^, CBS 18491, CBS 18500 and CBS 18501 did not show any morphological differences. After seven days of growth on GYPA at 25 °C, colonies are white, smooth, butyrous and entire and no pseudo-hyphae or hyphae are observed. However, slide cultures onto GYPA medium showed pseudohyphae after 4 days at 25 °C and after 8 days at 37 °C (Fig. 2a, b). After 3 days on GYPA at 25 °C, cells are subglobose to ovoid, 2–5 × 3–5.5 μm, and occur singly or in pairs (Fig. 2c). Reproduction took place by multilateral budding (Fig. 2). In addition, ascospore formation was not observed when mixing all strains on the sporulation media at 25 °C for up to 18 weeks.

Strains CBS 18490, CBS 18491, CBS 18500 and CBS 18501 were all able to grow at 37 °C, but no growth was observed at 40 °C, similarly to what has been reported for the type strains of the including (pathogenic) sibling species (Table 1). The four strains were all able to assimilate *N*-acetyl-glucosamine, cadaverine, ethylamine, galactose, d-glucose, glycerol, l-lysine, d-mannitol, melezitose, methyl-α-d-glucoside, pepton, potassium gluconate, raffinose, d-saccharose (sucrose), soluble starch (d-maltose), d-sorbitol, l-sorbose, and d-trehalose (Table 1). There was no specific compound that could or could not be assimilated by CBS 18490, CBS 18491, CBS 18500 and CBS 18501, compared to its close relatives (Table 1). To compare the various strains and species we collected phenotypic data of the type strains CBS 14366^T^, CBS 7798^T^, CBS 10004^T^, CBS 5149^T^, CBS 12666^T^, CBS 10701^T^, CBS 10913^T^, and CBS 16213^T^, from previous studies and the CBS Culture Collection database [9, 10, 21–23].

**Table 1:** Overview of phenotypic characteristics of the *Candidozyma molenica* strains compared to the type strains of sibling species

	*C. vulturna*	*C. chanthaburiensis*	*C. duobushaemuli*	*C. pseudohaemuli*	*C. haemuli*	*C. konsanensis*	*C. heveicola*	*C. auris*	*C. khanbhai*	*C. molenica*			
Phenotypic characteristic	CBS 14366^T^	CBS 10926^T^	CBS 7798^T^	CBS 10004^T^	CBS 5149^T^	CBS 12666^T^	CBS 10701^T^	CBS 10913^T^	CBS 16213^T^	CBS 18490^T^	CBS 18491	CBS 18500	CBS 18501
*Fermentation*													
d-Glucose	+	+	+	+	+	+	+	+	+	+	+	+	+
d-Galactose	–	–	+	+	–	–	–	–	–	–	–	–	–
Sucrose	+	–	+	–	+	+/w	+	w	+, s	+	+	+	+
Maltose	–	–	ND	ND	–	–	w/d	–	+/d	–	–	–	–
Lactose	–	–	ND	ND	ND	ND	ND	–	ND	–	–	–	–
Raffinose	+, w	–	+	–	–	–	ND	–	w/d	+	+	–	+
Trehalose	+	–	ND	ND	w/d	ND	ND	w	+	+	–	–	+
*Assimilation*													
Inulin	+	–	+/–	–	–	w	–	w	+	ND	ND	ND	ND
Raffinose	+	+	+	+	+	–	+	+	+	+	+	+	+
Melibiose	–	–	–/v	–	–	–	–	–	–/w	–	–	–	–
d-Galactose	+	+	+	+	+	+	+	–	+	+	+	+	+
Melezitose	+	+	+	+	+	+	+	+	+	+	+	+	+
Methyl-α-d-glucoside	w/d/+	w	–/w/d	v	–	+	w	–	+	+	+	+	+
Soluble starch (d-maltose)	+	+	+	+	+	+	–	+	+	+	+	+	+
Cellobiose	–/+	ND	–	–	–	ND	–	–	w	–	–	–	–
Salicin	–/w/d	+	–	–	–	–	+	–	–	ND	ND	ND	ND
l-Sorbose	–	+	+	d	–	+	+	–	+	+	+	+	+
l-Rhamnose	+	+	+	+	+	+	–	–	w/d	+	+	–	+
d-Xylose	–/w/d	+	d	d	d	w	d	–	+	w	w	–	–
l-Arabinose	+	+	–/w	v	–	d	–	–	+	–	–	–	–
d-Arabinose	–,w	–	d	–	–	w	ND	–	w	ND	ND	ND	ND
d-Ribose	–	+	–/w	+	d	d	–	–	+	w	w	–	+
Methanol	–	–	w/d	–	w/d	–	–	–	–	ND	ND	ND	ND
Ethanol	–	–	w/d	d	d	d	+	–	–	ND	ND	ND	ND
*meso*-Erythritol	–	w	–/d	–	–	–	–	–	w/d	–	–	–	–
Ribitol	+	s	+	+	d	+	+	w	+	ND	ND	ND	ND
Xylitol	+	ND	+	+	w/d	–	ND	–	+	ND	ND	ND	ND
Galactitol	ND	+	ND	+	ND	+	+	+	+	ND	ND	ND	ND
*myo*-Inositol	–	–	–/w	–	–	–	–	–	–	–	–	–	–
Gluconolactone	+	+	+	+	+	+	ND	ND	+	ND	ND	ND	ND
2-Keto-d-gluconate	ND	+	ND	+	ND	+	ND	–	+	+	+	+	+
dl-Lactate	–	–	–/w/d	ND	–	–	–	–	–	–	–	–	–
Succinate	+	+	+	+	+	+	+	–	+	ND	ND	ND	ND
Citrate	+	+	+	+	+	+	+	+	–	ND	ND	ND	ND
d-Gluconate	+	+	+	+	+	+	ND	–	+	ND	ND	ND	ND
d-Glucosamine	+	+	+	+	–	+	+	–	+	+	+	+	+
d-Saccharose (Sucrose)	+	+	ND	+	+	ND	+	ND	+	+	+	+	+
*N*-Acetyl-Glucosamine	+	+	ND	ND	+	ND	ND	ND	+	+	+	+	+
d-Trehalose	+	+	ND	+	+	ND	+	ND	+	+	+	d/+	+
d-Mannitol	+	+	ND	+	+	ND	+	ND	+	+	+	+	+
d-Lactose	+	–	ND	ND	+	ND	ND	ND	–	–	–	–	–
d-Sorbitol	+	+	ND	ND	+	ND	ND	ND	+	+	+	+	+
Glycerol	+	+	ND	ND	+	ND	ND	ND	+	+	+	+	+
Sodium glucoronate	ND	–	ND	ND	ND	ND	ND	ND	–	–	–	–	–
Potassium gluconate	ND	–	ND	ND	ND	ND	ND	ND	+	+	+	+	+
d-Glucose	+	+	ND	ND	+	ND	ND	ND	+	+	+	+	+
Nitrate	w	–	–	–	–	–	–	–	–	–	–	–	–
Nitrite	w	+	–	–	–	–	–	–	–	–	–	–	–
Ethylamine	–	+	+	+	+	+	+	–	+	+	+	+	+
Cadaverine	–	+	+	+	+	+	+	+	+	+	+	+	+
d-Tryptophan	+	ND	ND	ND	–	ND	ND	ND	–	–	–	–	–
Pepton	ND	ND	ND	ND	ND	ND	ND	ND	+	+	+	+	+
l-Lysine	+	+	ND	ND	+	ND	ND	ND	+	+	+	+	+
d-Glucosamine HCl	+	ND	ND	ND	+	ND	ND	ND	–	–	–	–	–
*Growth on YM agar at*													
4 °C	ND	ND	ND	ND	ND	ND	ND	ND	w	ND	ND	ND	ND
10 °C	ND	ND	ND	ND	ND	ND	ND	ND	s	ND	ND	ND	ND
30 °C	+	+	+	+	+	+	+	+	+	+	+	+	+
37 °C	+	+	+	+	+	+	+	+	+	+	+	+	+
40 °C	ND	–	–	+	–	–	–	+	+	–	–	–	–
42 °C	ND	–	–	–	–	–	–	w/s	w/s	ND	ND	ND	ND
*Other phenotypic tests*													
Tolerance to cycloheximide 0.01% w/v	–	–	+	ND	+	+	ND	–	+	+	+	+	+

*d* delayed positive; *s* slowly positive; *v* variable; *w* weak positive; *ND* not determined

Phenotypic data of type strains CBS 14366^T^, CBS 7798^T^, CBS 10004^T^, CBS 5149^T^, CBS 12666^T^, CBS 10701^T^, CBS 10913^T^, and CBS 16213^T^, was collected from the CBS Culture Collection (https://www.wi.knaw.nl/) and from De Jong et al. [9], Limtong and Yongmanitchai [21], Satoh et al. [22], Sipiczki and Mohd Tap [10], and Wang et al. [23]. Note that over time various physiology methods were used

**Fig. 3:**
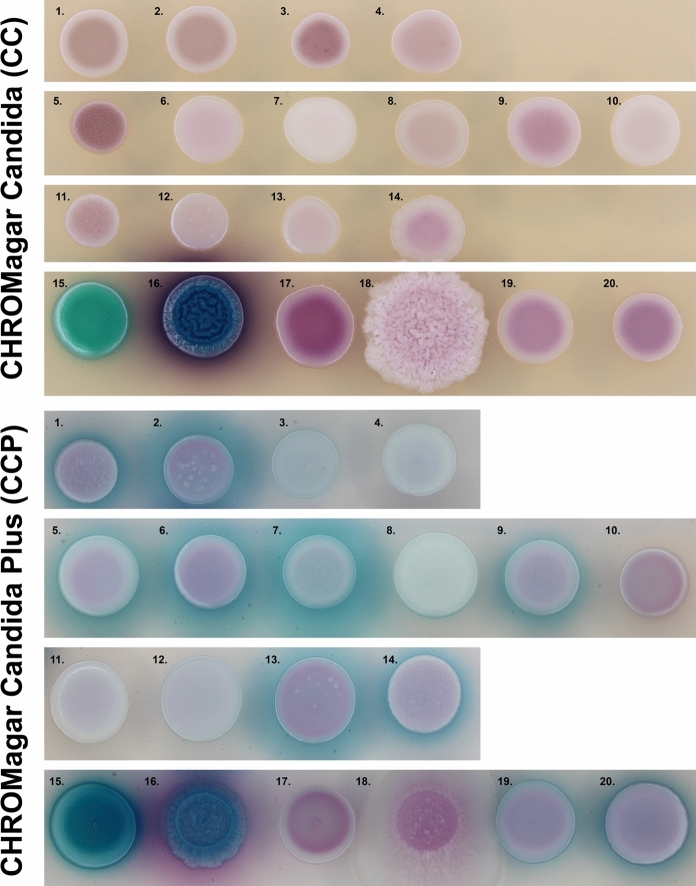
Growth of *Candidozyma molenica* and sibling species onto CHROMagar culture media. *Candidozyma molenica* strains and sibling species were inoculated onto CHROMagar Candida and CHROMagar Candida Plus culture media and incubated at 35 °C for 48 h. Numbers next to the colonies refer to the following species and strain numbers: 1–4 = *C. molenica* CBS 18490^T^, CBS 18491, CBS 18500, and CBS 18501; 5 = *C. haemuli* CBS 5149^T^; 6 = *C. pseudohaemuli* CBS 10004^T^; 7 = *C. duobushaemuli* CBS 7798^T^; 8 = *C. auris* CBS 10913^T^; 9 = *C. vulturna* CBS 14366^T^; 10 = *C. khanbhai* CBS 16213^T^; 11 = *C. heveicola* CBS 10701^T^; 12 = *C. chanthaburiensis* CBS 10926^T^; 13 = *C. konsanensis* CBS 12666^T^; 14 = *C. ruelliae* CBS 10815^T^; 15 = *C. albicans* CBS 562^NT^; 16 = *C. tropicalis* CBS 94^T^; 17 = *N. glabratus* CBS 138^T^; 18 = *P. kudriavzevii* CBS 573^T^; 19 = *C. parapsilosis* CBS 604^T^; 20 = *C. orthopsilosis* CBS 10906^T^

Onto CHROMagar Candida (CC) and CHROMagar Candida Plus (CCP) the *C. molenica* strains did show differences onto the latter. The two strains from clinical specimens (CBS 18490^T^ and CBS 18491) had a light purple colony with a blue halo in the CCP medium, while the two environmental strains (CBS 18500 and CBS 18501) had a white colony phenotype on CCP medium (Fig. 3). Hence, *C. molenica* can phenotypically resemble several pathogenic yeast species, like *C. haemuli*, *C. duobushaemuli*, *C. pseudohaemuli*, *C. vulturna*, but also the two non-clinical species *C. konsanensis* and *C. ruelliae*. Moreover, it can also be misidentified with this medium as *C. parapsilosis*, *C. orthopsilosis*, or even *C. auris* (Fig. 3).

### Antifungal Susceptibility Testing

Table 2 summarizes the observed minimum inhibitory concentrations (MIC’s) for the nine tested antifungal compounds for each of the four tested strains. From this, the fluconazole MIC’s for the two clinical-derived strains stand out compared to the two flower-associated strains, as the latter two had MIC’s of 8 mg/L compared to 1 mg/L for the former two. The azole compounds itraconazole, voriconazole and isavuconazole MIC’s were elevated for the two flower-associated strains CBS 18500 and CBS 18501 compared to the clinical-derived strains CBS 18490 and CBS 18491. The MIC for posaconazole was higher (0.06 mg/L) for CBS 18500, while the other three strains had a MIC of ≤ 0.015 mg/L. The MIC’s for the echinocandins did not vary much, all within one twofold dilution step, with 0.03 or 0.06 mg/L for anidulafungin and 0.06 or 0.125 mg/L for micafungin.

**Table 2:** Antifungal susceptibility minimum inhibitory concentrations of nine antifungal compounds

Antifungal	CBS 18490^T^ MIC (mg/L)	CBS 18491 MIC (mg/L)	CBS 18500 MIC (mg/L)	CBS 18501 MIC (mg/L)
Amphotericin B	1	1	2	2
Flucytosine	≤ 0.125	≤ 0.125	≤ 0.125	≤ 0.125
Fluconazole	1	1	8	8
Itraconazole	≤ 0.03	≤ 0.03	0.25	0.25
Voriconazole	≤ 0.03	≤ 0.03	0.125	0.125
Posaconazole	≤ 0.015	≤ 0.015	0.06	≤ 0.015
Isavuconazole	≤ 0.03	≤ 0.03	0.125	0.06
Anidulafungin	0.06	0.03	0.06	0.06
Micafungin	0.125	0.125	0.06	0.06

*MIC* Minimum inhibitory concentration

### Description of *Candidozyma molenica* A. Zandijk, T. van der Bruggen, M. Groenew. and F. Hagen sp. nov.

MycoBank number: MB 857463.

*Candidozyma molenica* (*molenica* refers to the hospital ward named ‘Molen’, Dutch for ‘mill’, where the first strain was obtained from a pediatric oncology patient).

Growth on GYPA after 3 days at 25 °C, the cells were subglobose to ovoid, 2–5 × 3–5.5 μm, and occur singly or in pairs. After growth at 25 °C for seven days colonies are white, smooth, butyrous and entire. Slide cultures showed pseudohyphae formation at 25 °C and 37 °C after 4 days and 8 days on GYPA incubation, respectively. No sexual spores are observed on the sporulation media tested after 2 months of growth at 25 °C. Carbon compounds assimilated are d-galactose, sucrose, *N*-acetyl glucosamine, d-raffinose, maltose, d-trehalose, potassium-2-keto-d-gluconate, d-methyl-α-d-glucopyranoside, d-mannitol, d-sorbitol, d-xylose (delayed/weak), d-glycerol, l-rhamnose (variable), d-melezitose, potassium gluconate, d-glucose, l-sorbose and glucosamine. Carbon compounds not assimilated are lactic acid, l-arabinose, d-cellobiose, d-lactose, inositol, erythritol, d-ribose, ethylamine, d-melibiose and d-glucuronate. Nitrogen compounds assimilated are ethylamine, l-lysine and cadaverine, and pepton. Nitrogen compounds not assimilated are nitrite, nitrate, d-glucosamine HCl and tryptophan. Fermentation of d-glucose, sucrose, raffinose (variable), and trehalose (variable) is positive. Growth occurred at 15 °C to 37 °C and is absent at 40 °C. Growth was observed in the presence of 0.01% cycloheximide.

Holotype: CBS 18490 was isolated from a patient in September 2023 in Utrecht, The Netherlands and preserved in a metabolically inactive state at the CBS culture collection hosted at the Westerdijk Fungal Biodiversity Institute, Utrecht, The Netherlands. Ex-type: 2MG-A1904-10.

Additional strains: CBS 18491, isolated from the same patient in September 2023 in Utrecht, The Netherlands, CBS 18500 and CBS 18501, both isolated from flowers, in 2006 in Belize City, Belize.

## Discussion

In this study we introduced the species *Candidozyma molenica* that belongs to the *Candidozyma haemuli* species complex. The strains came from flowers and from a throat and rectal swab of a pediatric oncology patient. This patient seems to be (tempory) colonized with *C. molenica* but this yeast did not cause an infection, although the four *C. molenica* strains were found to be capable of growing at 37 °C (Table 1). Phylogenetic analyses showed that the *C. molenica* strains could not be discerned from each other, and that these strains form a highly supported genetic lineage using different phylogenetic analyses (Fig. 1). Although the phylogenetic analyses resulted in high support values for majority of *Candidozyma* species, a small set of previously recognized species (*C. duobushaemuli*, *C. metrosideri* and *C. vulturna*) that cluster together had moderate to low support with all phylogenetic approaches (Fig. 1). In routine clinical diagnostics, the use of the ITS ribosomal DNA locus is the preferred approach for sequence-based identification of pathogenic fungi [15]. Although it is a reliable tool for species identification, including the members of the *C. haemuli* species complex, it cannot be used to discriminate between the recognized clades of *C. auris* [24]. Given the high support for *C. molenica* in the phylogenetic analyses we foresee that *C. molenica* can be reliably identified based on the ITS marker only, as applies for all other (pathogenic) species in this species complex (Fig. 1).

Interestingly, *C. molenica* is most closely related to species that have so far only been isolated from environmental sources (viz. *C. chanthaburiensis*, *C. heveicola*, *C. konsanensis*, and *C. ohialehuae*). Some of these ‘environmental’ species are able to grow at 37 °C, but are more distantly related to the well-known human pathogenic species within the *C. haemuli* species complex (viz. *C. pseudohaemuli*, *C. vulturna*, *C. duobushaemuli*, and *C. haemuli*) (Fig. 1; [25]). *Candidozyma molenica* might become the exception as a potential human pathogen when compared to its nearest sibling species that have not yet been described from human sources. So far, *C. molenica* has not been isolated from an invasive infection but as it can survive at human body temperature it fulfills one of the requirements of a human fungal pathogen, as it also can produce pseudohyphae at this temperature (Fig. 2b) it may be possible that *C. molenica* can invade human tissue.

Extensive phenotypic characterization showed that there was no single compound that could or could not be assimilated by *C. molenica* strains when compared to type strains of sibling species (Table 1). Remarkably, is the observation of phenotypic differences onto CCP medium of the two environmental strains versus the two strains that were obtained from clinical specimens. The latter appears as light rose colonies surrounded by a blue halo, while the environmental strains had a dull white colony phenotype (Fig. 3). However, onto CC medium no phenotypic difference was observed. Noteworthy is that the use of CCP is in a diagnostic laboratory preferred above CC as it can better discriminate pathogenic *Candida* species from each other [26, 27]. Chromogenic media are a valuable tool in a diagnostic setting, however, it has the drawback that some major pathogenic *Candida* species resemble a similar colony appearance as rare species, or even species that have a more resistant phenotype [28–30]. Hence, confirmation with another more laborious and/or expensive identification tool, such as MALDI-TOF MS or sequencing, is often needed. So far, not all species of the *C. haemuli* species complex are available in the commercial MALDI-TOF MS databases, leading in the past to ‘no identification possible’ or misidentification of the multi-drug resistant *C. auris* as other less-resistant pathogenic yeasts [31]. Hence, to correctly identify *C. molenica* by MALDI-TOF MS, we created a reference profile to distinguish it from *C. pseudohaemuli*, with which it was initially misidentified (see Data Availability). Such an approach of an in-house database has been recently used to identify other pathogenic and non-pathogenic species in the *C. haemuli* species complex [9, 32].

Another important characteristic of members of the *C. haemuli* species complex is their decreased susceptibility to one or more of the clinically used antifungal compounds amphotericin B, azoles, and echinocandins [33–35]. The four *C. molenica* strains had MIC values which is for all tested antifungal compounds in the lower range of what has been reported for pathogenic members of the *C. haemuli* species complex [34]. For the azoles, most *C. molenica* strains had low MIC values for itraconazole, voriconazole, posaconazole, and isavuconazole. However, the fluconazole MIC values stand out as the two environmental strains, CBS 18500 and CBS 18501, had higher MIC values of 8 mg/L compared to an MIC of 1 mg/L for the two strains from clinical specimen (CBS 18490^T^ and CBS 18491). Notably, also the MIC values for amphothericin B, itraconazole, voriconazole, posaconazole, isavuconazole, anidulafungin and micafungin, were all slightly higher for the two environmental strains (Table 1). For most of these MIC values these differences are within a two-fold dilution and this variation is within the allowed range. It is, however, remarkable that both environmental strains have slightly increased MIC values, indicating that these *C. molenica* strains might have been exposed to antifungal compounds. It remains enigmatic if this difference in antifungal susceptibility, although within the two-fold dilution range, is substantiated by (epi)genetic differences between the clinically derived and the environmental strains of *C. molenica*. For this, in-depth genome sequencing and expression experiments are needed to unravel the complexity of antifungal resistance. Such data is, however, lacking for majority of the species in the *C. haemuli* species complex, although the sudden global emergence of *C. auris* in clinical settings had caused an increased interest to study these resistance mechanisms compared to those of the major fungal pathogens [36].

## Data Availability

The ITS and D1/D2 ribosomal DNA sequences used for phylogenetic analyses are available via NCBI Nucleotide database, the accession numbers are listed in Fig. 1. Until *C. molenica* is part of the official Bruker MALDI-TOF MS reference profile database, the in-house created profile for *C. molenica* can be obtained on request from the corresponding author.
